# Post-ICU Care Trajectories and Outcomes Among Veterans: Comparing Veterans Affairs and Community Hospital Discharges

**DOI:** 10.1016/j.chest.2025.10.001

**Published:** 2025-10-17

**Authors:** Zachary Hahn, Hiam Naiditch, Martha F. Brucato, Victor Talisa, Brian Tyler, John R. Hotchkiss, Sachin Yende, Bryan J. McVerry, Florian B. Mayr

**Affiliations:** a VA Maine Togus Medical Center, Augusta, ME; b VA Pittsburgh Healthcare System, Critical Care Service Line, Pittsburgh, PA; c Division of Pulmonary, Allergy, Critical Care and Sleep Medicine, Department of Medicine, University of Pittsburgh School of Medicine, Pittsburgh, PA; d Clinical Research, Investigation, and Systems Modeling of Acute Illness (CRISMA) Center, University of Pittsburgh School of Medicine, Pittsburgh, PA; e Office of Integrated Veteran Care, Veterans Health Administration, Washington, DC

**Keywords:** Bayesian mediation analysis, community care, critical illness, health care trajectories, post-ICU follow-up, veterans

## Abstract

**Background:**

Survivors of critical illness face fragmented care transitions, leading to readmissions, emergency care use, and death. The Veterans Affairs (VA) Patient Aligned Care Team model emphasizes coordinated outpatient follow-up, yet many veterans now receive VA-purchased intensive care at community hospitals, potentially disrupting continuity.

**Research Question:**

How do outcomes after ICU care differ for veterans discharged from VA medical centers vs community hospitals, and what recovery trajectories emerge after discharge?

**Study Design and Methods:**

We conducted a retrospective cohort study of 311,224 veterans discharged home after medical ICU admission (2016-2023) using VA Corporate Data Warehouse and VA-purchased care claims. Outcomes included primary care visits, emergency department (ED) use, readmissions, mortality, and hospital-free days. Fine-Gray subdistribution hazard models estimated 30-day and 90-day risks, accounting for competing risks and adjusting for demographics, comorbidities, and facility clustering. Marginal standardization yielded adjusted risks, risk differences, and hazard ratios. A Bayesian mediation analysis tested whether 30-day primary care follow-up mediated readmission risk. Weekly transitions across 5 states—home, primary care visit, ED visit, readmission, and death—were analyzed with state sequence clustering.

**Results:**

Of 311,224 survivors of ICU stays, 36.9% were discharged from VA hospitals and 63.1% were discharged from community hospitals. At 30 days, patients from VA hospitals showed higher primary care follow-up rates (20.2% vs 15.3%), but higher 90-day readmission rates (27.0% vs 24.4%) and mortality rates (8.4% vs 7.3%). Patients discharged from community hospitals relied more on ED visits (32.1% vs 25.7%). Mediation analysis showed early primary care was protective, but explained little of the VA-community difference. Trajectory clustering revealed diverse recovery patterns, ranging from high primary care with low readmission to recurrent acute care use or early death.

**Interpretation:**

Our results showed that patients discharged from VA hospitals received earlier primary care, but showed higher readmission and mortality, whereas patients discharged from community hospitals made fewer visits but more ED use, highlighting the need for structured, risk-stratified transitional care.

## Graphical Abstract

**Figure:**
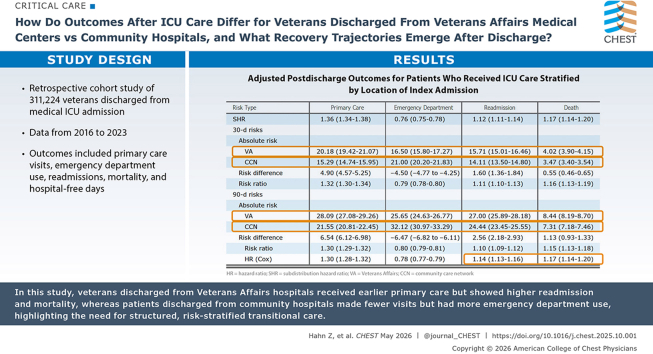

FOR EDITORIAL COMMENT, SEE PAGE 1159

**Take-Home Points**

**Research Question:** Among veterans surviving an ICU stay, how do postdischarge care trajectories and outcomes differ between those discharged from Veterans Affairs (VA) medical centers and those discharged from community hospitals?

**Results:** State-sequence clustering identified 5 distinct post-ICU trajectories, revealing that VA discharges had higher early primary care follow-up but greater readmission and mortality, whereas community discharges had fewer primary care visits and more emergency department use.

**Interpretation:** Our results show that post-ICU recovery among veterans is heterogeneous, and while VA-integrated primary care promotes earlier follow-up, both VA and community systems exhibit major transitional care gaps, underscoring the need for structured post-ICU programs beyond routine primary care visits.

Each year, > 5.7 million Americans are admitted to ICUs for syndromes such as sepsis or acute respiratory failure.^1^ Although most survive to discharge, recovery is marked by unresolved medical issues, new functional limitations, and exacerbations of chronic illness.^2^, ^3^, ^4^ Fragmented care transitions further heighten risks, contributing to delays in follow-up care, increased emergency department (ED) use, readmissions, and death.^5^^,^^6^ Many survivors and caregivers describe current care models as inadequate,^7^ illustrated by the fact that only 37.5% of Medicare enrollees discharged after acute respiratory failure see a primary care provider (PCP) within 60 days,^8^ and 40% of patients who survive to be discharged from the ICU are readmitted within 90 days.^9^

The Veterans Affairs (VA) Health Care System Patient-Aligned Care Team model offers vertically integrated primary care that could mitigate these challenges.^10^ However, recent policy changes have expanded access to community hospitals, a mix of private, nonprofit, and academic institutions with variable resources.^11^ This raises concerns about care continuity as more veterans receive ICU care outside the VA.

Evidence that structured follow-up reduces after ICU use is mixed,^12^ likely because of population heterogeneity and the one-size-fits-all approach of such interventions.^13^, ^14^, ^15^, ^16^ Whether early PCP follow-up can reduce adverse outcomes in real-world practice and whether the VA’s integrated system confers an advantage remain uncertain. Therefore, we conducted a national cohort study to examine whether veterans discharged from VA Medical Centers (VAMCs) showed higher PCP follow-up rates than those discharged from community hospitals and whether early PCP follow-up was associated with lower readmission rates and emergent care use. Furthermore, we used novel state sequence clustering to identify distinct post-ICU recovery phenotypes, aiming to clarify which patient subgroups are most vulnerable and how interventions could be targeted more effectively.

## Study Design and Methods

### Study Design and Data Sources

We conducted a retrospective cohort study in accordance with the Strengthening the Reporting of Observational Studies in Epidemiology Statement guidelines. Data were obtained from the VA Corporate Data Warehouse for demographic, clinical, and health care use variables.^17^^,^^18^ Community hospital encounters were identified from VA-purchased care claims.^19^^,^^20^ Community hospital characteristics were linked via National Provider Identifiers to RAND Corporation hospital data.^21^

### Study Population

We included veterans discharged home after a medical ICU admission at VAMCs or community hospitals between January 1, 2016, and December 31, 2023, with 90-day follow-up through March 31, 2024. ICU admissions were identified using VA ICU bed codes^22^ and ICU-related revenue codes for community hospitals.^23^ Only the first ICU admission per patient was included. Surgical admissions were excluded, given distinct severity and recovery needs.^24^^,^^25^ Veterans were required to have an established VA affiliation (Patient-Aligned Care Team assignment) before admission. Veterans discharged to skilled nursing facilities, rehabilitation centers, long-term acute care hospitals, or hospice care were excluded.^26^^,^^27^

### Outcomes

We examined post-ICU care trajectories across 3 domains: PCP follow-up, unplanned acute care use (ED or urgent care visits, hospital readmissions), and mortality. These measures reflect key indicators of care continuity, health care use, and patient outcomes.^28^ We analyzed outcomes at 30 days and 90 days after discharge, representing clinically relevant time frames for short-term recovery and care transitions.^29^^,^^30^ Hospital-free days at 90 days were included as a patient-centered metric.^31^

### PCP Follow-Up

PCP follow-up was defined as an outpatient encounter with a VA-assigned Patient-Aligned Care Team provider or an equivalent generalist in the community (e-Table 1). Only patients whose first contact after hospital discharge was a PCP visit were classified as having direct outpatient follow-up.

### Unplanned Acute Care Use

ED or urgent care visits and hospital readmissions were captured separately. We further classified readmissions using Agency for Healthcare Research and Quality Prevention Quality Indicators to identify ambulatory care sensitive conditions as markers of potentially preventable hospitalizations.^32^

### Mortality Ascertainment

Mortality was ascertained from VA vital status files integrating Social Security Administration, Medicare, VA inpatient deaths, state death certificates, and National Cemetery records.^18^

### Time-to-Event Models

We used Fine-Gray subdistribution hazards models to estimate the association between hospital type (VA vs community hospital) and outcomes after discharge at 30 and 90 days, accounting for outcome-specific competing risks.^33^^,^^34^ Adjusted absolute risks, risk differences (RDs), and risk ratios were obtained using marginal standardization over the study population.^35^^,^^36^ Cause-specific Cox proportional hazards models were fit as secondary analysis, treating competing events as censored, to provide complementary etiologic estimates.^37^

### Covariates and Sensitivity Analyses

Covariates were selected based on prior literature and were informed by directed acyclic graphs illustrating hypothesized relationships among hospital type, patient characteristics, length of stay (LOS), and outcomes (e-Fig 1).^38^, ^39^, ^40^ Models adjusted for age, sex, race, ethnicity, marital status, rurality, Area Deprivation Index, Charlson Comorbidity Index, diagnosis-related group (DRG) weight, ICU LOS, and hospital LOS. Because ICU and hospital LOS may serve as mediators or colliders influenced by unmeasured acute severity, we conducted sensitivity analyses excluding these variables. Physiologic severity scores were unavailable in administrative data; Charlson Comorbidity Index and DRG weight were used as proxies for illness severity.

### Mediation Analyses

We conducted a Bayesian mediation analysis to test whether 30-day PCP follow-up mediated the association between hospital type and 90-day readmission risk. We fit 2 linked Bernoulli regression models within a joint multivariate framework implemented using the Bayesian Regression Modeling via Stan (brms) package: (1) the probability of PCP follow-up as a function of hospital type (VA vs community) and (2) the probability of 90-day readmission as a function of hospital type, PCP follow-up, a hospital type by PCP interaction, and covariates. Models were estimated in brms with weakly informative priors and the No-U-Turn Sampler.^29^^,^^41^ Facility-level clustering was modeled with random intercepts for hospital identifiers. Natural direct and indirect effects (including both community and VA-indexed natural indirect effects because of the inclusion of the interaction) were obtained by applying posterior draws to counterfactual exposure-mediator scenarios. Convergence was confirmed with R-hat values, adequate effective sample sizes, and posterior predictive checks. Results are reported as absolute RDs with 95% credible intervals (CrIs).

### State Sequence and Clustering Analysis

To identify distinct care trajectories after discharge, we constructed weekly state sequences over 13 weeks, ranking health states by increasing acuity: home, PCP visit, ED visit, readmission, and death (absorbing state). When multiple events occurred in the same week, the highest-acuity state was assigned.^38^, ^39^, ^40^ State-sequence clustering was selected because it preserves the sequence and timing of care events, allowing detection of clinically meaningful patterns that frequency-based summaries or conventional survival models cannot capture. Distances between sequences were computed using the longest common subsequence matrix, which quantifies similarity in both structure and timing of events. Partitioning around medoids clustering with frequency weighting then was applied to minimize bias from duplicate sequences. The number of clusters was determined by optimizing the average silhouette width and assessing clinical interpretability (e-Table 2).^42^ Clusters were characterized further using an overrepresentation index (OI), which measures whether a specific patient group is represented disproportionately within a given cluster relative to its prevalence in the overall cohort.^43^

All statistical analyses were conducted using R version 4.1.1 software (R Foundation for Statistical Computing). We summarized baseline characteristics using descriptive statistics. We report continuous variables as mean (SD) or median (interquartile range [IQR]) and categorical variables as frequencies. Statistical significance was defined as a 2-sided *P* value of < .05. No adjustments were made for multiple comparisons. Additional details on model specifications, coding definitions, clustering procedures, and sensitivity analyses are provided in e-Appendix 1.

### Ethical Review

The Veterans institutional review board Network of Northern New England (Identifier: 1760529) and VA Pittsburgh institutional review board (Identifier: 1742737) approved this study with a waiver of informed consent.

## Results

### Baseline Characteristics

We analyzed 311,224 veterans discharged after medical ICU admission between 2016 and 2023, including 114,756 patients (36.9%) at VAMCs and 196,468 patients (63.1%) at community hospitals (e-Fig 2). Community hospitals were more likely to be rural and less likely to be teaching facilities compared with VAMCs (e-Table 3).

Table 1 summarizes baseline characteristics. The median age was 70 years (IQR, 62-76 years) in both groups, with similar Charlson Comorbidity Index scores. VA patients were more likely to be Black (n = 27,703 [24.1%] vs n = 32,334 [16.5%]) or Hispanic (n = 7,071 [6.2%] vs n = 8,851 [4.5%]) and were less often White (n = 79,435 [69.2%] vs n = 146,950 [74.7%]) or married (n = 52,806 [46.0%] vs n = 99,574 [50.7%]) compared with community patients. Rural residence was more common among community patients (n = 78,421 [39.9%] vs n = 33,799 [29.5%]). VA patients showed a higher prevalence of cardiac disease (n = 33,635 [27.6%] vs n = 45,138 [22.7%]) and other common comorbidities (Table 1). They also experienced a longer median hospital LOS (5 days [IQR, 3-9 days] vs 3 days [IQR, 2-5 days]), but lower DRG weights (median, 1.06 [IQR, 0.80-1.50] vs 1.17 [IQR, 0.88-1.67]). The top discharge diagnoses were similar between VA and community discharges (e-Table 4).

**Table 1:** Veteran Characteristics Stratified by Index Admission Location

Variable	All Veterans (N = 311,224)	VAMC Group (n = 114,756)	CCN Group (n = 196,468)
Age, y	70 (62-76)	70 (63-75)	70 (62-76)
Male sex	294,097 (94.5)	109,039 (95.0)	185,058 (94.2)
Race			
White	226,385 (72.6)	79,435 (69.2)	146,950 (74.7)
Black	60,037 (19.3)	27,703 (24.1)	32,334 (16.5)
Asian	1,523 (0.5)	498 (0.4)	1,025 (0.5)
Other^a^	6,101 (2.0)	1,854 (1.6)	4,247 (2.2)
Unknown	17,178 (5.5)	5,266 (4.6)	11,912 (6.1)
Ethnicity			
Hispanic	15,992 (5.1)	7,071 (6.2)	8,851 (4.5)
Non-Hispanic	283,001 (90.9)	103,913 (90.5)	179,088 (91.2)
Unknown	12,301 (4.0)	3,772 (3.3)	8,529 (4.3)
Married	152,380 (49.0)	52,806 (46.0)	99,574 (50.7)
Rural residence	112,220 (36.1)	33,799 (29.5)	78,421 (39.9)
Area Deprivation Index	62 (41-81)	61 (39-81)	62 (42-81)
Charlson Comorbidity Index	3 (1-5)	3 (1-6)	3 (1-5)
Cerebrovascular disease	53,904 (17.3)	20,769 (18.1)	33,135 (16.9)
Cardiac disease	76,353 (24.5)	33,635 (27.6)	45,138 (22.7)
Pulmonary disease	112,739 (36.2)	42,739 (37.2)	70,000 (35.6)
Chronic kidney disease	64,771 (20.8)	25,187 (21.9)	39,584 (20.1)
Diabetes	142,527 (45.8)	53,821 (46.9)	88,706 (45.2)
Chronic liver disease	38,989 (12.5)	16,901 (13.9)	23,088 (11.8)
Malignancy	58,739 (18.9)	25,003 (21.8)	33,736 (17.2)
Dementia	16,659 (5.4)	6,521 (5.7)	10,138 (5.2)
Admission source			
Outpatient	240,298 (77.2)	111,137 (96.8)	129,161 (65.7)
Interfacility transfer	21,727 (7.0)	3,137 (2.7)	18,590 (9.5)
Other or Unknown	49,199 (15.8)	482 (0.4)	48,717 (24.8)
DRG weight	1.15 (0.85-1.63)	1.06 (0.80-1.50)	1.17 (0.88-1.67)
LOS			
ICU	2 (1-4)	2 (1-3)	2 (1-4)
Hospital	4 (2-7)	5 (3-9)	3 (2-5)

Data are presented as No. (%) or median (interquartile range). CCN = community care network; DRG = diagnosis-related Group; LOS = length of stay; VAMC = Veterans Affairs Medical Center.

a Includes races other than White, Black, or Asian.

### Unadjusted Outcome Rates

PCP follow-up was higher after VA discharge (30-day, 20.5% vs 15.6%; 90-day, 28.0% vs 21.9%). ED use was higher after community discharge (30-day, 21.7% vs 16.2%; 90-day, 31.7% vs 26.8%). Readmissions were higher after VA discharge (30-day, 16.2% vs 13.9%; 90-day, 28.1% vs 23.9%). Mortality also was higher after VA discharge (30-day, 4.4% vs 3.3%; 90-day, 8.8% vs 7.2%). About 1 in 5 readmissions involved ambulatory care-sensitive conditions (VA, 18.2%; community, 19.5%) (e-Table 5).

### Modeled Outcomes

Table 2 presents the results of the adjusted time to event modeling of outcomes with competing risk. In Fine-Gray competing risk models, VA patients were more likely to have a PCP follow-up within 30 days (subdistribution hazard ratio, 1.36; 95% CI, 1.34-1.38). Absolute 30-day probabilities were 20.2% for VA patients vs 15.3% for community patients (RD, 4.9%; 95% CI, 4.6%-5.3%), with a wider gap at 90 days (RD, 6.5%; 95% CI, 6.1%-7.0%). ED use was less common among VA patients at 30 days (16.5% vs 21.0%; RD, –4.5%; 95% CI, –4.8% to –4.3%). This difference increased further by 90 days (RD, –6.5%; 95% CI, –6.8% to –6.1%). Readmissions at 30 days were modestly more frequent after VA discharge (15.7% vs 14.1%; RD, 1.6%; 95% CI, 1.4%-1.8%), with only a small increase in RD from 30 to 90 days (RD, 2.6%; 95% CI, 2.2%-2.9%).

**Table 2:** Adjusted Postdischarge Outcomes for Patients Who Received ICU Care Stratified by Location of Index Admission

Risk Type	Primary Care	Emergency Department	Readmission	Death
SHR	1.36 (1.34-1.38)	0.76 (0.75-0.78)	1.12 (1.11-1.14)	1.17 (1.14-1.20)
30-d risks				
Absolute risk				
VA	20.18 (19.42-21.07)	16.50 (15.80-17.27)	15.71 (15.01-16.46)	4.02 (3.90-4.15)
CCN	15.29 (14.74-15.95)	21.00 (20.20-21.83)	14.11 (13.50-14.80)	3.47 (3.40-3.54)
Risk difference	4.90 (4.57-5.25)	–4.50 (–4.77 to –4.25)	1.60 (1.36-1.84)	0.55 (0.46-0.65)
Risk ratio	1.32 (1.30-1.34)	0.79 (0.78-0.80)	1.11 (1.10-1.13)	1.16 (1.13-1.19)
90-d risks				
Absolute risk				
VA	28.09 (27.08-29.26)	25.65 (24.63-26.77)	27.00 (25.89-28.18)	8.44 (8.19-8.70)
CCN	21.55 (20.81-22.45)	32.12 (30.97-33.29)	24.44 (23.45-25.55)	7.31 (7.18-7.46)
Risk difference	6.54 (6.12-6.98)	–6.47 (–6.82 to –6.11)	2.56 (2.18-2.93)	1.13 (0.93-1.33)
Risk ratio	1.30 (1.29-1.32)	0.80 (0.79-0.81)	1.10 (1.09-1.12)	1.15 (1.13-1.18)
HR (Cox)	1.30 (1.28-1.32)	0.78 (0.77-0.79)	1.14 (1.13-1.16)	1.17 (1.14-1.20)

SHRs were estimated using Fine-Gray competing risk models, with CCN as the reference group. These models account for competing events (eg, death) and were adjusted for age, sex, race, ethnicity, marital status, rurality, Area Deprivation Index, Charlson Comorbidity Index, diagnosis-related group weight, hospital and ICU lengths of stay, and admission location. HRs were estimated using cause-specific Cox models, treating competing events as censored. Absolute risks, risk differences (VA minus CCN), and risk ratios (VA divided by CCN) are presented at 30 and 90 days with 95% CIs. CCN = community care network; HR = hazard ratio; SHR = subdistribution hazard ratio; VA = Veterans Affairs.

VA discharges had higher hazards of death (subdistribution hazard ratio, 1.17; 95% CI, 1.14-1.20). Absolute risks were 4.0% vs 3.5% at 30 days (RD, 0.55%; 95% CI, 0.46%-0.65%) and 8.4% vs 7.8% at 90 days. Full model specifications, coefficients, and sensitivity analyses are provided in e-Tables 6-11. Cox models were directionally consistent with Fine-Gray estimates.

### Mediation Analysis

VA admission was associated with a higher probability of 30-day PCP follow-up (absolute RD, 6.31%; 95% CrI, 5.82%-7.70%). The protective association of PCP follow-up with 90-day readmission differed by hospital type, with an estimated reduction of –4.40% (95% CrI, –4.60% to –3.81%) among community patients and –3.10% (95% CrI, –3.36% to –2.34%) among VA patients, yielding an interaction contrast of –1.29% (95% CrI, –1.63% to –0.33%). Because the outcome model included this interaction, 2 versions of the natural indirect effect were identified: community-indexed natural indirect effects, comparing community patients if the PCP follow-up occurred at VA rates vs community rates (–0.28%; 95% CrI, –0.36% to –0.22%), and VA-indexed natural indirect effects, comparing VA patients if the PCP follow-up occurred at VA rates vs community rates (–0.20%; 95% CrI, –0.27% to –0.14%). Both indirect effects were small compared with the pure natural direct effect of VA admission (3.28%; 95% CrI, 2.58%-3.98%); the total effect was 3.08% (95% CrI, 2.38%-3.77%).

All Bayesian models converged, and posterior checks confirmed fit (e-Fig 3, 4). Sensitivity analyses (no VA × PCP interaction, excluding ICU LOS, wider priors) yielded similar effects (e-Table 12); regression estimates and diagnostics are summarized in e-Table 13.

### Trajectory Clustering and State Sequencing Analysis

**Figure 1:**
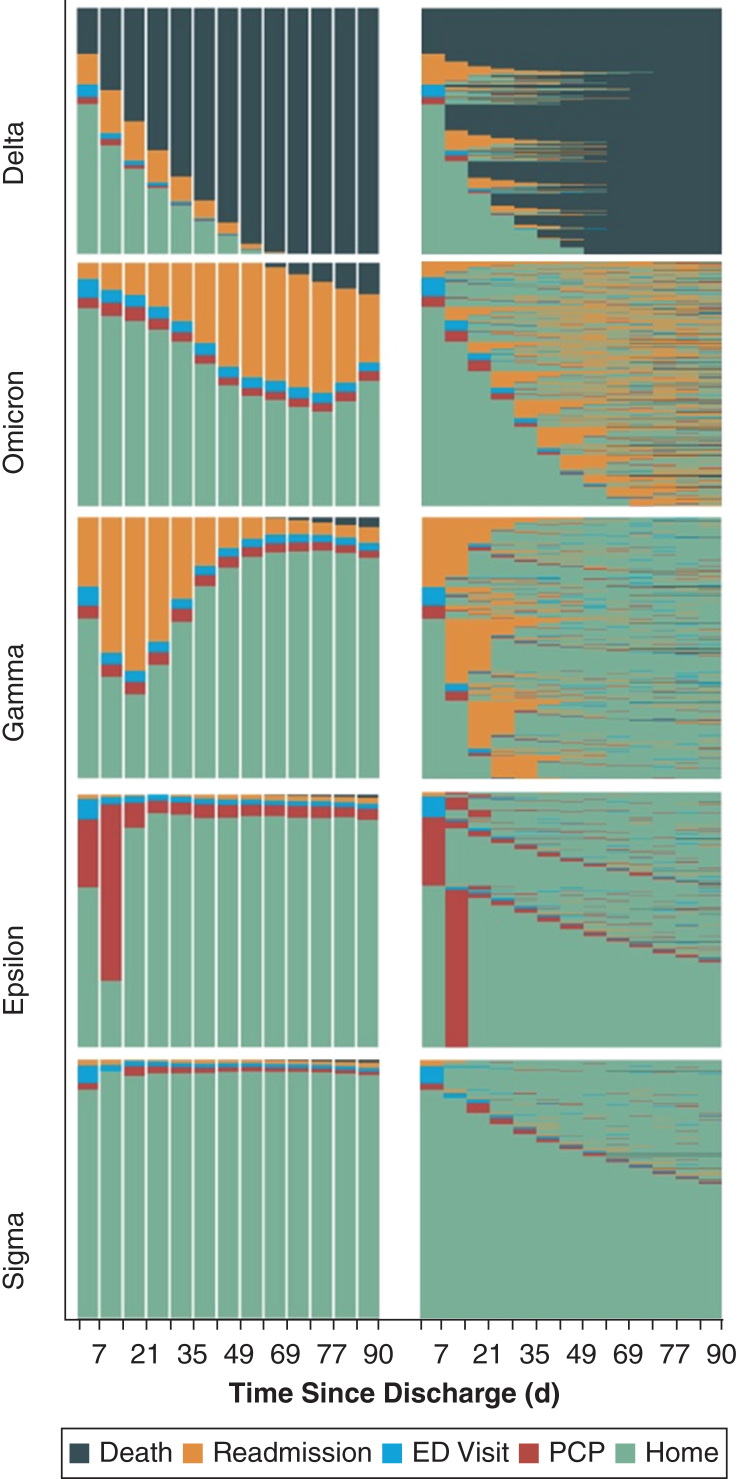
Weekly state density plots by cluster. Each row represents a distinct postdischarge trajectory cluster—Delta, Omicron, Gamma, Epsilon, and Sigma—identified through state sequence analysis. The left column displays weekly state density plots, illustrating the proportion of patients occupying each postdischarge state (home, PCP visit, ED visit, readmission, and death) over time. The right column presents index plots, showing individual-level transitions between states, with each horizontal line representing a patient’s trajectory through 90 days after discharge. Key patterns emerge across clusters. For example, Delta shows a rapid transition to death with minimal outpatient engagement, whereas Sigma remains predominantly at home with limited health care contact. ED = emergency department; PCP = primary care provider.

**Figure 2:**
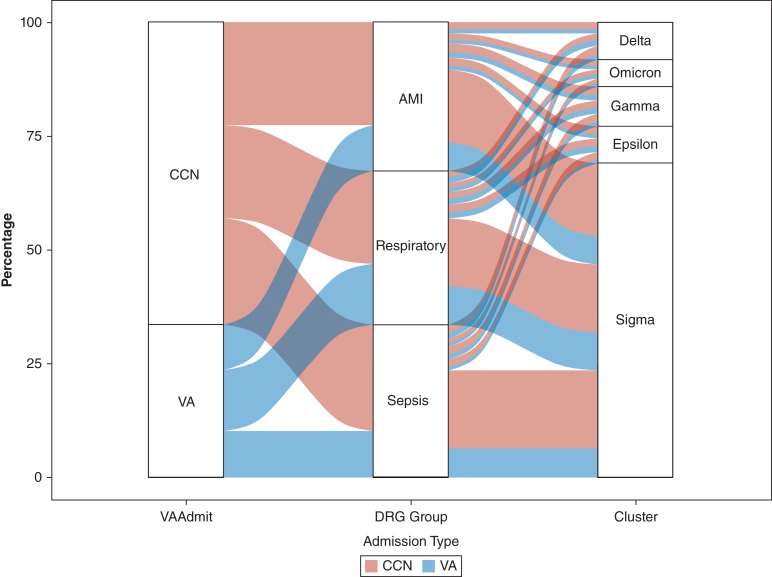
Flow diagram showing postdischarge care trajectories by DRG. This figure illustrates the movement of veterans from initial hospitalization through major DRG categories (eg, AMI, respiratory, sepsis) into 5 distinct postdischarge trajectory clusters (Delta, Omicron, Gamma, Epsilon, and Sigma). The visualization highlights how different diagnostic groups contribute to varying postdischarge pathways, reflecting differences in health care use and recovery patterns. DRG definitions: sepsis (DRG 870-872), acute respiratory failure (DRG 003, 004, 207, 208, and 189-195), and AMI (DRG 223-225, 280-285, and 291-293). AMI = acute myocardial infarction; CCN = community care network; DRG = diagnosis-related group; VA = Veterans Affairs.

State sequence clustering identified 5 distinct trajectories after ICU care illustrating heterogeneous recovery patterns and imbalances in patient flow between VA and community admissions (Fig 1, Table 3). Additionally, common ICU diagnoses, including sepsis, acute myocardial infarction, and acute respiratory failure, exhibited distinct outpatient trajectories that varied based on the location of index admission (Fig 2, e-Table 14).

**Table 3:** Patient Characteristics and 90-Day Outcomes by Trajectory Cluster

Characteristic	Delta (n = 18,376)	Omicron (n = 15,576)	Gamma (n = 24,214)	Epsilon (n = 24,498)	Sigma (n = 228,560)
Age, y	74 (69-82)	70 (63-76)	70 (63-76)	69 (62-75)	70 (61-75)
Male sex	17,851 (0.97)	14,900 (0.96)	23,047 (0.95)	23,077 (0.94)	215,222 (0.94)
VAMC admission	7,764 (0.42)	6,832 (0.44)	10,563 (0.44)	9,732 (0.40)	79,865 (0.35)
Charlson Comorbidity Index	4 (2-7)	4 (2-7)	4 (2-6)	3 (1-5)	3 (1-5)
DRG weight	1.48 (1.14-1.85)	1.21 (0.91-1.67)	1.22 (0.93-1.67)	1.06 (0.81-1.48)	1.08 (0.84-1.60)
Urban residence	11,223 (0.61)	10,360 (0.67)	15,702 (0.65)	15,309 (0.62)	144,975 (0.63)
ADI	63 (42-82)	63 (42-82)	62 (41-81)	60 (40-79)	61 (41-81)
LOS, d					
Hospital	6 (4-10)	5 (3-8)	5 (3-8)	4 (2-6)	4 (2-6)
ICU	3 (2-5)	3 (2-4)	3 (2-4)	2 (1-4)	2 (1-4)
PCP follow-up at 90 d	1,672 (9.1)	3,824 (24.6)	3,920 (16.2)	21,902 (89.4)	43,723 (19.1)
No. of PCP visits at 90 d	0 (0-0)	0 (0-1)	0 (0-1)	1 (1-2)	1 (0-1)
Emergent care use at 90 d	6,067 (33.0)	9,730 (62.5)	13,806 (57.0)	7,619 (31.1)	55,821 (24.4)
No. of ED visits at 90 d	1 (0,1)	1 (1-3)	1 (0-2)	0 (0-1)	1 (0-1)
Readmissions at 90 d	7,655 (41.7)	15,346 (98.5)	23,454 (96.9)	3,320 (13.6)	29,403 (12.9)
No. of readmissions at 90 d	1 (1-1)	2 (1-3)	1 (1-2)	0 (0-0)	0 (0-1)
Death at 90 d	18,376 (100)	2,012 (12.9)	921 (3.8)	295 (1.2)	2,623 (1.1)
Time to death, d	23 (9-39)	73 (66-81)	75 (68-83)	75 (67-83)	73 (64-82)
Hospital-free days at 90 d	19 (7-35)	85 (81-87)	84 (78-87)	90 (89-90)	90 (89-90)

Data are presented as No. (%) or median (interquartile range). This table provides an overview of patient characteristics and 90-day outcomes across 5 distinct post-ICU care trajectory clusters: Delta, Omicron, Gamma, Epsilon, and Sigma. Key characteristics such as age, sex, VAMC admission rate, Charlson Comorbidity Index, DRG weight, urban residence, and hospital and ICU LOS are compared among the clusters. Additionally, the table presents health care use patterns and outcomes, including PCP follow-up and visit frequency, emergent care use, number of ED visits, readmissions, number of readmissions, mortality rates, time to death, and hospital-free days at 90 days. Trends indicate differences in acute illness severity, outcomes, and health care use patterns among the clusters. This comparison highlights the distinctive clinical trajectories and resource use patterns associated with different clusters. ADI = Area Deprivation Index; DRG = diagnosis-related group; ED = emergency department; LOS = length of stay; PCP = primary care provider; VAMC = Veterans Affairs Medical Center.

The Delta cluster (n = 18,376) was characterized by rapid clinical deterioration, with 100% mortality by 90 days. These patients typically were older (median age, 74 years; IQR, 69-82 years), with high comorbidity burden and elevated DRG weights. PCP follow-up at 90 days was rare (9.1%), and the median time to death was 23 days (IQR, 9-39 days). Patients with sepsis admitted to VAs were overrepresented (global OI, 1.16; within-DRG OI, 1.29).

The Omicron cluster (n = 15,576) exhibited the highest acute care use. Nearly all patients (98.5%) experienced at least 1 readmission (median, 2 readmissions; IQR, 1-3 readmissions), ED visits were frequent (median, 1 ED visit; IQR, 1-3 ED visit), and 90-day mortality was 12.9%. Patients admitted to VA hospitals were represented disproportionately in this cluster for all 3 common ICU diagnoses.

The Gamma cluster (n = 24,214) showed a pattern of progressive clinical decline. Most patients were readmitted (96.9%), and more than one-half made an ED visit (57%). PCP follow-up was low (16.2%), and 90-day mortality was 3.8%. Patients with acute respiratory failure admitted to VAs were overrepresented (global OI, 1.37; within-DRG OI, 1.17).

The Epsilon cluster (n = 24,498) was defined by early and sustained primary care engagement. PCP follow-up occurred in 89.4% of patients, ED use was infrequent (31.1%; median, 0 visits; IQR, 0-1 visit), and outcomes were favorable, with 90-day readmission at 13.6% and mortality at 1.2%. Patients admitted to VA hospitals were represented disproportionately in this cluster for all 3 ICU diagnoses.

The Sigma cluster (n = 228,560), the largest trajectory, was characterized by low health care engagement. Only 19.1% had PCP follow-up, 24.4% made an ED visit, and the 90-day readmission rate was 12.9%. Patients admitted to community hospital were overrepresented across all 3 diagnostic groups.

## Discussion

In this national cohort of > 300,000 veterans, we observed that recovery trajectories after ICU care varied substantially by discharge location and patient subgroup. Veterans discharged from VAMCs were more likely to engage with primary care, yet experienced higher readmission and mortality risks, whereas those discharged from community hospitals showed lower outpatient follow-up and greater reliance on emergency care. State sequence clustering further demonstrated that recovery was not uniform, but instead followed distinct patterns of outpatient engagement, acute care use, and survival.

Our mediation analysis adds nuance to these observations. VA admission increased the probability of 30-day PCP follow-up by approximately 6%. Early PCP visits were associated with reduced 90-day readmission risk in both the community and VA cohorts, although the protective effect was somewhat stronger among the community cohort. Yet, in causal decomposition, both the cross-world and true natural indirect effects were small, whereas the direct effect of VAMC admission on readmissions persisted. These results indicate that although primary care engagement is modestly protective, it cannot account fully for the higher readmission burden among VA patients. In the context of prior randomized trials and systematic reviews showing inconsistent effects of post-ICU clinics,^7^^,^^13^^,^^15^ our findings reinforce that uniform follow-up strategies are unlikely to be sufficient in heterogeneous survivor populations.

Trajectory clustering extends these insights by identifying clinically relatable phenotypes that clarify which survivors are most vulnerable and which interventions are most effective. Veterans in the Delta group, who experienced rapid mortality, underscore the importance of systematic goals-of-care discussions and earlier integration of serious-illness care. The Omicron and Gamma groups, marked by recurrent acute care use despite outpatient contact, highlight the limitations of routine primary care and the potential value of structured post-ICU or disease-specific clinics. By contrast, the Epsilon group demonstrated the protective association of reliable primary care engagement, whereas the large Sigma group revealed a population effectively lost to follow-up, pointing to the need for re-engagement strategies such as automatic PCP scheduling, telehealth bridging visits, or navigator outreach. These recovery phenotypes illustrate why prior interventions after ICU care may have yielded inconsistent results: not because follow-up is irrelevant, but because interventions were not targeted to the patients most likely to benefit. Our current analysis could not predict cluster membership reliably using administrative data alone. This represents an important avenue for future research. Incorporating more granular data may enable accurate risk stratification at the time of discharge. Developing predictive tools to identify prospectively patients at risk for trajectories such as Delta, Omicron, or Sigma would allow clinicians to match interventions at the time of discharge, addressing a central limitation of prior post-ICU trials.

The distribution of these phenotypes also reflects the broader systems in which veterans receive care, emphasizing the role of policy, geography, and institutional capacity in shaping recovery after critical illness. The decision to pursue follow-up within VA or community systems is influenced by distance, availability, and perceptions of care quality.^44^ Community hospitals often serve dispersed, rural populations with fewer local resources. These constraints can make timely follow-up more challenging to achieve, despite best efforts, and may explain the overrepresentation of community discharges in the Sigma phenotype. Nearly 1 in 5 readmissions involved ambulatory care-sensitive conditions, consistent with prior reports.^45^ This highlights how gaps in chronic disease management and care coordination contribute to morbidity after ICU discharge. Strengthening timely primary and specialty follow-up therefore may reduce a substantial proportion of readmissions, particularly among patients belonging to high-use (Omicron and Gamma) and underengaged (Sigma) phenotypes. With ongoing policy shifts, exemplified by the Maintaining Internal Systems and Strengthening Integrated Outside Networks Act of 2018 (MISSION) Act, the Sigma trajectory may expand unless deliberate efforts are made to strengthen referral pathways and to ensure continuity of care back into the VA. Without proactive efforts, preventable readmissions, including those for ambulatory care-sensitive conditions, may rise, particularly among disengaged veterans in the Sigma phenotype. Taken together, these findings emphasize that trajectories after ICU care are shaped not only by clinical characteristics, but also by system-level structures and policies.

Several limitations should be acknowledged. Our use of administrative data precluded adjustment for key confounders, including functional status, frailty, social determinants, and patient preferences. Although we adjusted for comorbidity burden and DRG weight, physiologic severity scores (eg, Sequential Organ Failure Assessment and Acute Physiology and Chronic Health Evaluation) were not available, so residual confounding by acute illness severity may remain. Differences in diagnostic coding practices between VA and community hospitals also may affect risk adjustment, because VAMCs do not face reimbursement incentives tied to DRG assignment. Consequently, case complexity may be underestimated, which could inflate observed readmission and mortality rates.^46^^,^^47^

In addition, our analysis was limited to VA-delivered and VA-purchased care, which may underestimate the health care received outside these systems. Community hospitals are highly heterogeneous, and although we attempted to capture their variation in size, ICU capacity, teaching status, and safety-net designation, these measures do not reflect fully the variation in clinical practice or coordination. We further limited the cohort to patients discharged home, so our findings may not apply to veterans discharged to skilled nursing, rehabilitation, or long-term acute care facilities.

More broadly, the COVID-19 pandemic likely altered discharge patterns and outpatient access during the latter study years. Finally, our cohort was predominantly male, which limits the generalizability of the results to nonveteran and more sex-diverse populations.

## Interpretation

Veterans experience markedly different recovery trajectories after ICU discharge. Patients discharged from VAMCs were associated with greater primary care engagement, but also higher readmission and mortality risk, whereas patients discharged from community hospitals were marked by lower outpatient follow-up and greater reliance on emergency care. Although early PCP follow-up was modestly protective, it explained only a small portion of the VA-community difference in outcomes, reinforcing that primary care alone is insufficient. Our findings underscore the importance of risk-stratified approaches, which involve integrating serious-illness care for veterans at risk of early mortality, developing structured transitional or disease-specific follow-up for those with recurrent acute care use, and implementing systematic re-engagement strategies for underengaged populations. At the policy level, expanding community care without deliberate continuity safeguards may widen gaps; ensuring timely reconnection to VA services will be critical. Because routine primary care engagement within the VA did not fully reduce the risk of readmissions, future efforts must extend beyond existing models by testing and scaling targeted transitional care pathways through implementation science.

## Funding/Support

F. B. M. was supported by the 10.13039/100000057National Institute of General Medical Sciences, 10.13039/100000002National Institutes of Health [Grant K23GM132688]. This work is also supported by a VA Pittsburgh Eugene Marsh Pilot Award, additional resources provided by VA Pittsburgh Healthcare System, and the central data repositories maintained by the VA Information Resource Center, including the Corporate Data Warehouse.

## Financial/Nonfinancial Disclosures

None declared.

## Supplementary Data

e-Online Data
