# Supplementary material for: Post-ICU Care Trajectories and Outcomes Among Veterans: Comparing Veterans Affairs and Community Hospital Discharges
Source: Chest. 2025 Oct 17;169(5):1228–39. doi: 10.1016/j.chest.2025.10.001 (PMC13197970; doi:10.1016/j.chest.2025.10.001)
Supplement: e-Online Data [file mmc1.docx]

# Post-ICU Care Trajectories and Outcomes Among Veterans: Comparing VA and Community Hospital Discharges

Online-Only Supplement

[Supplemental Methods](#_Toc353842173)

[eTable 1. Codes Used to Identify Outpatient Encounter Types by Hospital Setting](#_Toc226437478)

[eTable 2. Cluster Quality Metrics by Number of Clusters (k)](#_Toc1296862315)

[eTable 3. Comparison of Hospital Characteristics: VAMC vs. Community Hospitals](#_Toc189223382)

[eTable 4. Top 25 Discharge-Related Groups (DRG) Codes for VAMC and CCN Admissions](#_Toc991930685)

[eTable 5. Overall Rates and Condition-Specific Distribution of ACSC Readmissions After ICU Discharge, by Admission Location](#_Toc1173736526)

[eTable 6. Fine-Gray Model Specifications and Coefficients for PCP Follow-up](#_Toc1109998771)

[eTable 7. Fine-Gray Model Specifications and Coefficients for 90-day Emergent Care Use](#_Toc1832584366)

[eTable 8. Fine-Gray Model Specifications and Coefficients for 90-day Readmissions](#_Toc2024887658)

[eTable 9. Cox Proportional Hazards Model Specifications and Coefficients for 90-day Mortality](#_Toc536736991)

[eTable 10. Sensitivity Analysis of Confidence Intervals for Calculated Risks Using Fine-Gray Model with M-out-of-N Nonparametric Bootstrapping](#_Toc2142599186)

[eTable 11. Sensitivity of Subdistribution Hazard Ratios to Exclusion of Hospital and ICU Length of Stay Covariates](#_Toc1720490364)

[eTable 12. Bayesian Mediation Analysis (Absolute Risk Difference in %)](#_Toc351278635)

[eTable 13. Posterior Regression Estimates and Convergence Diagnostics for Mediator (PCP30) and Outcome (Readmit90) Models](#_Toc739050552)

[eTable 14. Distribution and Overrepresentation of DRGs and Clusters in VAMC vs. CCN Discharges](#_Toc1868065799)

[eFigure1. Simplified Directed Acyclic Graph Frameworks Informing Covariate Selection and Sensitivity Analyses.](#_Toc298313722)

[eFigure 2. Cohort Selection Flowchart for ICU Survivors Discharged Home Between 2016 and 2023.](#_Toc729648279)

[eFigure 3. MCMC Trace Plots for Representative Mediator (PCP30) and Outcome (Readmit90) Parameters](#_Toc1047282504)

[eFigure 4. Decile-Binned Calibration of Predicted vs. Observed 90-day Readmission Probabilities](#_Toc1356332089)

# Supplemental Methods

## Competing Risk Model Specifications

Fine–Gray subdistribution hazards were estimated using the crr function from the ***cmprsk*** package in R.^1,2^ Competing events were defined according to the outcome of interest. For primary care follow-up, emergency department (ED) visits, hospital readmissions, or death were treated as competing events. For ED visits, the competing events were hospital readmissions or death, while primary care follow-up was not considered competing. For hospital readmission, the only competing event was death. Mortality, as the terminal outcome, was modeled using Cox proportional hazards with the coxph function from the ***survival*** package, which is equivalent to a Fine–Gray model without competing events.^3,4^ The time origin was hospital discharge, and patients were administratively censored at 90 days.

## Estimation of Absolute Risks

Adjusted absolute risks and contrasts were obtained by marginal standardization.^5^ For each fitted model, predicted risks were generated for every patient and then averaged across the observed covariate distribution to yield population-averaged estimates separately for VA and community discharges. This approach produced adjusted risks at 30 and 90 days, risk differences defined as VA minus community, and risk ratios defined as VA divided by community.

## Confidence Interval Estimation

Uncertainty for model-based subdistribution hazard ratios (SHRs) and hazard ratios (HRs) was derived from the model variance–covariance matrix. Confidence intervals for adjusted risks, risk differences, and risk ratios were obtained via parametric bootstrapping.^6^ Using the mvnorm function from the ***MASS*** package, we simulated 1,000 coefficient draws from a multivariate normal distribution defined by the original coefficient estimates and variance–covariance matrix.^7^ We then reported the 2.5th and 97.5th percentiles as the 95% confidence interval. As a sensitivity analysis, we conducted an m-out-of-n nonparametric bootstrap using random subsamples of 30,000 observations per iteration, repeated 1,000 times (eTable 10).^8^ For each iteration, we refit the model, recomputed adjusted risks and contrasts, and then derived percentile-based intervals. These nonparametric bootstrap results are presented in the supplement only.

## Secondary Analyses

Cause-specific Cox proportional hazards models were estimated for each non-mortality outcome, treating competing events as censoring.^9^ Hazard ratios from these models reflect the relative instantaneous risk of the event of interest among patients still at risk. Estimates were directionally and quantitatively consistent with the Fine–Gray results.

## Sensitivity Analyses

Because hospital and ICU length of stay may act as colliders in the relationship between unmeasured severity and outcomes, we performed sensitivity analyses in which these covariates were excluded. Results from these models are reported in eTable 11.

## Trajectory Analysis

Weekly post-discharge states were constructed over the 13 weeks following discharge, ordered by increasing clinical acuity as home, primary care visit, ED visit, hospital readmission, and death, which was modeled as an absorbing state. When multiple events occurred within the same week, the state corresponding to the highest acuity was assigned. Pairwise distances between state sequences were calculated using the longest common subsequence metric.^10^ Partitioning Around Medoids clustering with frequency weighting was then applied to avoid overrepresentation of duplicate sequences.^11^ Cluster composition was further described using an Overrepresentation Index, which quantifies whether particular subgroups of patients were disproportionately represented in a given cluster relative to their prevalence in the full cohort.^12^

To determine the optimal number of clusters, we compared candidate solutions ranging from two to six clusters. For each specification, we evaluated cluster quality indices including point biserial correlation, Hubert’s Gamma, average silhouette width, Calinski–Harabasz indices, and pseudo R².^12–14^ Two-cluster solutions maximized silhouette width and distance-reproduction metrics but merged clinically distinct recovery patterns into broad categories. Increasing the number of clusters modestly reduced certain fit statistics but improved clinical coherence by separating trajectories with high primary care engagement, recurrent acute care use, or rapid mortality. Sequence density plots supported these distinctions. The final clustering solution was therefore selected by balancing statistical validity with clinical interpretability (eTable 2).

## Primary Care Definition

VA primary care encounters were identified using Stop Codes for Patient Aligned Care Team providers. Community primary care encounters were defined by Current Procedural Terminology and National Provider Identifier taxonomy codes for internal medicine, family medicine, nurse practitioners, and physician assistants. This definition encompassed outpatient encounters with generalist providers delivering longitudinal medical care (eTable 1).

## Missing Data

Analyses used complete cases. The frequency of missing data was low and did not differ meaningfully by hospital type. Patients excluded due to missing covariates were similar in baseline characteristics to those included. Given the low and non-differential rate of missingness, imputation was not performed.

## Software and Reproducibility

All analyses were conducted in R version 4.1.1 using the ***cmprsk***, ***survival***, ***brms***, ***WeightedCluster***, and ***MASS*** packages (R Core Team, 2021).

## References

1. Fine JP, Gray RJ. A Proportional Hazards Model for the Subdistribution of a Competing Risk. J Am Stat Assoc. 1999;94(446):496-509. doi:10.1080/01621459.1999.10474144

2. Gray B. cmprsk: Subdistribution Analysis of Competing Risks. Published online May 19, 2024. Accessed September 10, 2025. https://cran.r-project.org/web/packages/cmprsk/index.html

3. Modeling Survival Data: Extending the Cox Model | SpringerLink. Accessed September 10, 2025. https://link.springer.com/book/10.1007/978-1-4757-3294-8

4. Therneau TM, until 2009) TL (original S >R port and R maintainer, Elizabeth A, Cynthia C. survival: Survival Analysis. Published online December 17, 2024. Accessed September 10, 2025. https://cran.r-project.org/web/packages/survival/index.html

5. Austin PC, Fine JP. Practical recommendations for reporting Fine-Gray model analyses for competing risk data. Stat Med. 2017;36(27):4391-4400. doi:10.1002/sim.7501

6. Efron B. Bootstrap confidence intervals for a class of parametric problems. Biometrika. 1985;72(1):45-58. doi:10.1093/biomet/72.1.45

7. Ripley B, Venables B. MASS: Support Functions and Datasets for Venables and Ripley’s MASS. Accessed September 10, 2025. http://www.stats.ox.ac.uk/pub/MASS4/

8. Bickel PJ, Götze F, Zwet WRV. Resampling fewer than n observations: Gains, losses, and remedies for losses. Stat Sin. 1997;7(1):1-31.

9. Gerds TA, Scheike TH, Andersen PK. Absolute risk regression for competing risks: interpretation, link functions, and prediction. Stat Med. 2012;31(29):3921-3930. doi:10.1002/sim.5459

10. Abbott A, Tsay A. Sequence Analysis and Optimal Matching Methods in Sociology. Sociol Methods Res. 2000;29(1):3-33. doi:10.1177/0049124100029001001

11. Partitioning Around Medoids (Program PAM). In: Finding Groups in Data. John Wiley & Sons, Ltd; 1990:68-125. doi:10.1002/9780470316801.ch2

12. Studer, Matthias (2013). WeightedCluster Library Manual: A practical guide to creating typologies of trajectories in the social sciences with R. LIVES Working Papers, 24. DOI: http://dx.doi.org/10.12682/lives.2296-1658. 2013.24

13. Caliński T, Harabasz J. A dendrite method for cluster analysis. Commun Stat. 1974;3(1):1-27. doi:10.1080/03610927408827101

14. Rousseeuw PJ. Silhouettes: A graphical aid to the interpretation and validation of cluster analysis. J Comput Appl Math. 1987;20:53-65. doi:10.1016/0377-0427(87)90125-

# eTable 1. Codes Used to Identify Outpatient Encounter Types by Hospital Setting

| Encounter Type | Setting | Codes Used | Description / Notes |
| --- | --- | --- | --- |
| Emergency Department | VA | Stop Code 130 (ED) | VA emergency encounters. |
|  | Community | POS 23 (Emergency Department);  CPT 99281–99285 (ED visits); CPT 99291–99292 (critical care) | Identified using place of service and CPT; critical care codes restricted to ED encounters (excluding inpatient). |
| Urgent Care | VA | Stop Code 131 (Urgent Care) | VA urgent care encounters |
|  | Community | POS 20 (Urgent Care);  CPT 99202–99205 (new); CPT 99211–99215 (established); CPT 99241–99245 (legacy consults) | Office/urgent care outpatient visits are billed in urgent care settings. Consultation codes included for residual use. |
| Primary Care | VA | Stop Codes: 170, 172, 301, 318, 322, 323, 342, 348, 350, 704, 323531 | PACT providers include general internal medicine, family practice, geriatrics, women’s care, and serious mental illness primary care. |
|  | Community | POS 11 (Office);  CPT 99202–99205 (new); CPT 99211–99215 (established); CPT 99241–99245 (legacy consults);  NPI Taxonomy Codes: 207Q00000X, 207QA0000X, 207QA0401X, 207QA0505X, 207QB0002X, 207QG0300X, 207QS0010X, 207R00000X, 207RA0000X, 207RA0002X, 207RB0002X, 208D00000X, 363LA2200X, 363LC1500X, 363LF0000X, 363LG0600X, 363LP2300X, 363LW0102X, 364SA2200X, 364SF0001X, 364SG0600X, 364SH0200X, 364SW0102X | Defined using CPT office/outpatient codes and provider taxonomy codes for family medicine, internal medicine, general practice, nurse practitioners, and clinical nurse specialists. |
| Legacy CPT consultation codes (99241–99245) and deleted office code 99201 were included to capture residual billing practices in community datasets. Place of Service (POS) codes were required to distinguish Office (11), Urgent Care (20), and Emergency Department (23) settings. | | | |

# eTable 2. Cluster Quality Metrics by Number of Clusters (k)

| k | PBC | HG | HGSD | ASW | ASWw | CH | R2 | CHsq | R2sq | HC |
| --- | --- | --- | --- | --- | --- | --- | --- | --- | --- | --- |
| 2 | 0.82 | 0.99 | 0.99 | 0.79 | 0.79 | 126,226.40 | 0.29 | 434,843.9 | 0.58 | 0.03 |
| 3 | 0.76 | 0.95 | 0.94 | 0.62 | 0.62 | 98,434.81 | 0.39 | 348,292.6 | 0.69 | 0.04 |
| 4 | 0.64 | 0.85 | 0.81 | 0.38 | 0.38 | 74,305.12 | 0.42 | 256,908.6 | 0.71 | 0.08 |
| 5 | 0.63 | 0.88 | 0.85 | 0.40 | 0.40 | 63,976.36 | 0.45 | 219,551.7 | 0.74 | 0.06 |
| 6 | 0.63 | 0.90 | 0.86 | 0.40 | 0.40 | 57,083.53 | 0.48 | 206,524.3 | 0.77 | 0.05 |
| Cluster Quality Metrics based on Studer M (2013):   - PBC (Point Biserial Correlation): [−1; 1] Max. Measure of the capacity of the clustering to reproduce the distances. - HG (Hubert’s Gamma): [−1; 1] Max. Measure of the capacity of the clustering to reproduce the distances (order of magnitude). - HGSD (Hubert’s Somers’ D): [−1; 1] Max. Measure of the capacity of the clustering to reproduce the distances (order of magnitude), taking into account ties in distances. - HC (Hubert’s C): [0; 1] Min. The gap between the partition obtained and the best partition theoretically possible with this number of groups and these distances. - ASW (Average Silhouette Width): [−1; 1] Max. Coherence of assignments. High coherence indicates high between-group distances and strong within-group homogeneity. - ASWw (Average Silhouette Width, weighted): [−1; 1] Max. As previously mentioned, for floating-point weights. - CH (Calinski-Harabasz index): [0; +∞[ Max. Pseudo F computed from the distances. - CHsq (Calinski-Harabasz index, squared): [0; +∞] Max. As previously, but using squared distances. - R2 (Pseudo R2): [0; 1] Max. Share of the discrepancy explained by the clustering solution (only to compare partitions with an identical number of groups). - R2sq (Pseudo R2, squared): [0; 1] Max. As previously, but using squared distances. | | | | | | | | | | |

# eTable 3. Comparison of Hospital Characteristics: VAMC vs. Community Hospitals

| Characteristic | CCN (N = 3,025) | VAMC (N = 100) |
| --- | --- | --- |
| Acute Care Beds | 134 (50, 266) | 81 (50, 127) |
| ICU Beds | 14 (6, 30) | 17 (10, 28) |
| Bed Size Category |  |  |
| <50 | 747 (25%) | 24 (24%) |
| 50-99 | 434 (14%) | 35 (35%) |
| 100-199 | 783 (26%) | 27 (27%) |
| 200-399 | 693 (23%) | 9 (9%) |
| 400-599 | 200 (6.6%) | 1 (1%) |
| e |  |  |
| 600+ | 168 (5.6%) | 2 (2%) |
| ICU Size Category |  |  |
| <10 Icu | 1,103 (36%) | 24 (24%) |
| 10-19 Icu | 707 (23%) | 32 (32%) |
| 20-39 Icu | 680 (22%) | 26 (26%) |
| 40+ Icu | 535 (18%) | 16 (16%) |
| Teaching Status |  |  |
| Non-teaching | 1,791 (59%) | 1 (1%) |
| Teaching | 1,234 (41%) | 97 (97%) |
| Unknown | 0 | 2 |
| Rural/Urban |  |  |
| Urban | 2,147 (71%) | 91 (91%) |
| Rural | 876 (29%) | 8 (8%) |
| Unknown | 2 | 1 |
| Safety-net (DSH>0) | 2,391 (79%) | - |
| Hospital characteristics of Veterans Affairs Medical Centers (VAMCs) and community hospitals. Characteristics include acute inpatient bed size, ICU bed size, teaching status (based on Graduate Medical Education [GME] funding), and urban/rural status (based on RUCA codes). Community hospital data are derived from RAND hospital files; VAMC data are derived from national VA datasets. | | |

# eTable 4. Top 25 Discharge-Related Groups (DRG) Codes for VAMC and CCN Admissions

**VAMC Admissions**

| DRG | DRG Description | Count | Percent | Cumulative |
| --- | --- | --- | --- | --- |
| 871 | Septicemia Or Severe Sepsis W/O Mv >96 Hours W Mcc | 8,002 | 7.0 | 7.0 |
| 189 | Pulmonary Edema & Respiratory Failure | 5,827 | 5.1 | 12.1 |
| 309 | Cardiac Arrhythmia & Conduction Disorders W Cc | 3,450 | 3.0 | 15.1 |
| 638 | Diabetes W Cc | 3,223 | 2.8 | 17.9 |
| 291 | Heart Failure & Shock W Mcc | 2,824 | 2.5 | 20.3 |
| 310 | Cardiac Arrhythmia & Conduction Disorders W/O Cc/Mcc | 2,666 | 2.3 | 22.7 |
| 378 | G.I. Hemorrhage W Cc | 2,634 | 2.3 | 25.0 |
| 177 | Respiratory Infections & Inflammations W Mcc | 2,581 | 2.3 | 27.2 |
| 280 | Acute Myocardial Infarction, Discharged Alive W Mcc | 2,258 | 2.0 | 29.2 |
| 281 | Acute Myocardial Infarction, Discharged Alive W Cc | 2,034 | 1.8 | 30.9 |
| 308 | Cardiac Arrhythmia & Conduction Disorders W Mcc | 1,989 | 1.7 | 32.7 |
| 897 | Alcohol/Drug Abuse Or Dependence W/O Rehabilitation Therapy W/O Mcc | 1,987 | 1.7 | 34.4 |
| 190 | Chronic Obstructive Pulmonary Disease W Mcc | 1,933 | 1.7 | 36.1 |
| 303 | Atherosclerosis W/O Mcc | 1,847 | 1.6 | 37.7 |
| 641 | Misc Disorders Of Nutrition, Metabolism, Fluids/Electrolytes W/O Mcc | 1,794 | 1.6 | 39.3 |
| 287 | Circulatory Disorders Except Ami, W Card Cath W/O Mcc | 1,770 | 1.5 | 40.8 |
| 193 | Simple Pneumonia & Pleurisy W Mcc | 1,676 | 1.5 | 42.3 |
| 872 | Septicemia Or Severe Sepsis W/O Mv >96 Hours W/O Mcc | 1,610 | 1.4 | 43.7 |
| 637 | Diabetes W Mcc | 1,436 | 1.3 | 44.9 |
| 305 | Hypertension W/O Mcc | 1,397 | 1.2 | 46.1 |
| 377 | G.I. Hemorrhage W Mcc | 1,356 | 1.2 | 47.3 |
| 208 | Respiratory System Diagnosis W Ventilator Support <96 Hours | 1,354 | 1.2 | 48.5 |
| 392 | Esophagitis, Gastroent & Misc Digest Disorders W/O Mcc | 1,231 | 1.1 | 49.6 |
| 916 | Allergic Reactions W/O Mcc | 1,198 | 1.0 | 50.6 |
| 065 | Intracranial Hemorrhage Or Cerebral Infarction W Cc Or Tpa In 24 Hrs | 1,180 | 1.0 | 51.6 |

**CCN Admissions**

| DRG | DRG Description | Count | Percent | Cumulative |
| --- | --- | --- | --- | --- |
| 871 | Septicemia Or Severe Sepsis W/O Mv >96 Hours W Mcc | 18,358 | 9.3 | 9.3 |
| 291 | Heart Failure & Shock W Mcc | 9,356 | 4.8 | 14.1 |
| 177 | Respiratory Infections & Inflammations W Mcc | 7,617 | 3.9 | 18.0 |
| 287 | Circulatory Disorders Except Ami, W Card Cath W/O Mcc | 4,921 | 2.5 | 20.5 |
| 189 | Pulmonary Edema & Respiratory Failure | 4,797 | 2.4 | 22.9 |
| 280 | Acute Myocardial Infarction, Discharged Alive W Mcc | 4,720 | 2.4 | 25.3 |
| 065 | Intracranial Hemorrhage Or Cerebral Infarction W Cc Or Tpa In 24 Hrs | 4,569 | 2.3 | 27.7 |
| 309 | Cardiac Arrhythmia & Conduction Disorders W Cc | 4,481 | 2.3 | 29.9 |
| 378 | G.I. Hemorrhage W Cc | 4,157 | 2.1 | 32.1 |
| 193 | Simple Pneumonia & Pleurisy W Mcc | 4,048 | 2.1 | 34.1 |
| 190 | Chronic Obstructive Pulmonary Disease W Mcc | 3,963 | 2.0 | 36.1 |
| 310 | Cardiac Arrhythmia & Conduction Disorders W/O Cc/Mcc | 3,850 | 2.0 | 38.1 |
| 872 | Septicemia Or Severe Sepsis W/O Mv >96 Hours W/O Mcc | 3,737 | 1.9 | 40.0 |
| 281 | Acute Myocardial Infarction, Discharged Alive W Cc | 3,348 | 1.7 | 41.7 |
| 308 | Cardiac Arrhythmia & Conduction Disorders W Mcc | 3,066 | 1.6 | 43.3 |
| 683 | Renal Failure W Cc | 2,828 | 1.4 | 44.7 |
| 638 | Diabetes W Cc | 2,815 | 1.4 | 46.1 |
| 286 | Circulatory Disorders Except Ami, W Card Cath W Mcc | 2,685 | 1.4 | 47.5 |
| 208 | Respiratory System Diagnosis W Ventilator Support <96 Hours | 2,481 | 1.3 | 48.8 |
| 312 | Syncope & Collapse | 2,297 | 1.2 | 49.9 |
| 682 | Renal Failure W Mcc | 2,270 | 1.2 | 51.1 |
| 897 | Alcohol/Drug Abuse Or Dependence W/O Rehabilitation Therapy W/O Mcc | 2,208 | 1.1 | 52.2 |
| 377 | G.I. Hemorrhage W Mcc | 2,189 | 1.1 | 53.3 |
| 064 | Intracranial Hemorrhage Or Cerebral Infarction W Mcc | 2,176 | 1.1 | 54.4 |
| 917 | Poisoning & Toxic Effects Of Drugs W Mcc | 2,172 | 1.1 | 55.5 |

# eTable 5. Overall Rates and Condition-Specific Distribution of ACSC Readmissions After ICU Discharge, by Admission Location

| Readmissions (n, %) | Community  (n = 38,337) | VA  (n = 31,348) |
| --- | --- | --- |
| All ACSC Readmissions | 7,490 (19.50) | 5,707 (18.20) |
| COPD/Asthma | 1,227 (16.38) | 1,052 (18.43) |
| Diabetes, Long-Term Complication | 337 (4.50) | 209 (3.66) |
| Diabetes, Short-Term Complication | 254 (3.39) | 204 (3.57) |
| Diabetic Amputation | 28 (0.37) | 1 (0.02) |
| Heart Failure | 3,776 (50.41) | 2,742 (48.05) |
| Hypertension | 288 (3.85) | 344 (6.03) |
| Pneumonia | 927 (12.38) | 530 (9.29) |
| Urinary Tract Infections | 377 (5.03) | 364 (6.38) |
| Uncontrolled Diabetes | 276 (3.68) | 261 (4.57) |
| ACSC readmissions were defined using AHRQ Prevention Quality Indicators. Values represent the number and percentage of all-cause readmissions that were classified as ACSCs, followed by the distribution of specific ACSC categories within those readmissions for Community Care and Veterans Affairs (VA) discharges. | | |

# eTable 6. Fine-Gray Model Specifications and Coefficients for PCP Follow-up

| Characteristic | N | SHR^1^ | 95% CI^1^ | p-value |
| --- | --- | --- | --- | --- |
| AdmitAge | 309,760 | 1.00 | 1.00, 1.00 | 0.032 |
| Gender |  |  |  |  |
| M | 292,704 | — | — |  |
| F | 17,056 | 1.02 | 0.99, 1.06 | 0.2 |
| Race |  |  |  |  |
| White | 225,317 | — | — |  |
| Black | 59,758 | 0.92 | 0.90, 0.94 | <0.001 |
| Asian | 1,513 | 0.94 | 0.85, 1.04 | 0.3 |
| Other | 23,172 | 0.94 | 0.92, 0.97 | <0.001 |
| Ethnicity |  |  |  |  |
| Non-Hispanic | 293,890 | — | — |  |
| Hispanic | 15,870 | 1.01 | 0.98, 1.04 | 0.6 |
| Rurality |  |  |  |  |
| Urban | 197,548 | — | — |  |
| Rural | 112,212 | 1.04 | 1.02, 1.06 | <0.001 |
| MaritalStatus |  |  |  |  |
| Unmarried | 158,031 | — | — |  |
| Married | 151,729 | 1.07 | 1.05, 1.08 | <0.001 |
| CharlsonScore | 309,760 | 0.99 | 0.99, 1.00 | <0.001 |
| DRGWeight | 309,760 | 0.92 | 0.91, 0.94 | <0.001 |
| HospitalDays | 309,760 | 1.00 | 0.99, 1.00 | <0.001 |
| ICUDays | 309,760 | 1.00 | 1.00, 1.00 | 0.016 |
| ADINationalRank | 309,760 | 1.00 | 1.00, 1.00 | <0.001 |
| VAAdmit | 309,760 | 1.36 | 1.34, 1.38 | <0.001 |
| ^1^SHR = Subdistribution Hazard Ratio, CI = Confidence Interval  Estimates are derived from Fine–Gray subdistribution hazard models with 90-day censoring, treating death, readmissions, and ED visits as competing risks. Models quantify the association between admission location (VA vs community [CCN]) and time to first outpatient primary care follow-up within 90 days of discharge. Subdistribution hazard ratios (SHRs) greater than 1 indicate a higher likelihood of follow-up, accounting for the presence of competing risks. Covariates include demographic characteristics, comorbidities, hospital and ICU length of stay, Diagnosis-Related Group (DRG) weight, Area Deprivation Index (ADI) national rank, and marital status. Reference categories are shown with em dashes (—). | | | | |

# eTable 7. Fine-Gray Model Specifications and Coefficients for 90-day Emergent Care Use

| Characteristic | N | SHR^1^ | 95% CI^1^ | p-value |
| --- | --- | --- | --- | --- |
| AdmitAge | 309,760 | 1.00 | 1.00, 1.00 | <0.001 |
| Gender |  |  |  |  |
| M | 292,704 | — | — |  |
| F | 17,056 | 1.02 | 0.99, 1.05 | 0.3 |
| Race |  |  |  |  |
| White | 225,317 | — | — |  |
| Black | 59,758 | 0.96 | 0.94, 0.97 | <0.001 |
| Asian | 1,513 | 0.89 | 0.81, 0.98 | 0.020 |
| Other | 23,172 | 0.97 | 0.95, 1.0 | 0.018 |
| Ethnicity |  |  |  |  |
| Non-Hispanic | 293,890 | — | — |  |
| Hispanic | 15,870 | 1.06 | 1.03, 1.09 | <0.001 |
| Rurality |  |  |  |  |
| Urban | 197,548 | — | — |  |
| Rural | 112,212 | 0.98 | 0.96, 0.99 | 0.002 |
| MaritalStatus |  |  |  |  |
| Unmarried | 158,031 | — | — |  |
| Married | 151,729 | 0.90 | 0.89, 0.91 | <0.001 |
| CharlsonScore | 309,760 | 1.04 | 1.03, 1.04 | <0.001 |
| DRGWeight | 309,760 | 0.94 | 0.93, 0.96 | <0.001 |
| HospitalDays | 309,760 | 1.00 | 1.00, 1.00 | <0.001 |
| ICUDays | 309,760 | 0.98 | 0.97, 0.98 | <0.001 |
| ADINationalRank | 309,760 | 1.00 | 1.00, 1.00 | 0.3 |
| VAAdmit | 309,760 | 0.76 | 0.75, 0.78 | <0.001 |
| ^1^SHR = Subdistribution Hazard Ratio, CI = Confidence Interval  Estimates are derived from Fine–Gray subdistribution hazard models with 90-day censoring, treating death and readmissions as a competing risk. Models quantify the association between admission location (VA vs community[CCN]) and time to first emergent care use (emergency department visit or unplanned hospitalization) within 90 days of discharge. Subdistribution hazard ratios (SHRs) greater than 1 indicate a higher likelihood of emergent care use, accounting for the presence of competing risks. Covariates include demographic characteristics, comorbidities, hospital and intensive care unit (ICU) length of stay, Diagnosis-Related Group (DRG) weight, Area Deprivation Index (ADI) national rank, and marital status. Reference categories are shown with em dashes (—). | | | | |

# eTable 8. Fine-Gray Model Specifications and Coefficients for 90-day Readmissions

| Characteristic | N | SHR^1^ | 95% CI^1^ | p-value |
| --- | --- | --- | --- | --- |
| AdmitAge | 309,760 | 1.00 | 1.00, 1.01 | <0.001 |
| Gender |  |  |  |  |
| M | 292,704 | — | — |  |
| F | 17,056 | 0.92 | 0.89, 0.95 | <0.001 |
| Race |  |  |  |  |
| White | 225,317 | — | — |  |
| Black | 59,758 | 0.92 | 0.90, 0.94 | <0.001 |
| Asian | 1,513 | 0.90 | 0.81, 1.00 | 0.058 |
| Other | 23,172 | 0.94 | 0.91, 0.97 | <0.001 |
| Ethnicity |  |  |  |  |
| Non-Hispanic | 293,890 | — | — |  |
| Hispanic | 15,870 | 0.96 | 0.93, 0.99 | 0.015 |
| Rurality |  |  |  |  |
| Urban | 197,548 | — | — |  |
| Rural | 112,212 | 0.93 | 0.92, 0.95 | <0.001 |
| MaritalStatus |  |  |  |  |
| Unmarried | 158,031 | — | — |  |
| Married | 151,729 | 0.90 | 0.88, 0.91 | <0.001 |
| CharlsonScore | 309,760 | 1.08 | 1.08, 1.09 | <0.001 |
| DRGWeight | 309,760 | 1.06 | 1.05, 1.07 | <0.001 |
| HospitalDays | 309,760 | 1.01 | 1.01, 1.01 | <0.001 |
| ICUDays | 309,760 | 1.00 | 1.00, 1.00 | 0.018 |
| ADINationalRank | 309,760 | 1.00 | 1.00, 1.00 | <0.001 |
| VAAdmit | 309,760 | 1.12 | 1.11, 1.14 | <0.001 |
| ^1^SHR = Subdistribution Hazard Ratio, CI = Confidence Interval  Estimates are from Fine–Gray subdistribution hazard models with 90-day follow-up, treating death as a competing event. Models evaluate the association between admission location (VA vs community[CCN]) and time to first readmission within 90 days of discharge. Subdistribution hazard ratios (SHRs) greater than 1 indicate a higher likelihood of readmission, accounting for the influence of competing events. Covariates include demographic characteristics, comorbidities, hospital and intensive care unit (ICU) length of stay, Diagnosis-Related Group (DRG) weight, Area Deprivation Index (ADI) national rank, and marital status. Reference categories are denoted by em dashes (—). | | | | |

# eTable 9. Cox Proportional Hazards Model Specifications and Coefficients for 90-day Mortality

| Characteristic | N | HR^1^ | 95% CI^1^ | p-value |
| --- | --- | --- | --- | --- |
| AdmitAge | 309,760 | 1.05 | 1.05, 1.05 | <0.001 |
| Gender |  |  |  |  |
| M | 292,704 | — | — |  |
| F | 17,056 | 0.86 | 0.80, 0.93 | <0.001 |
| Race |  |  |  |  |
| White | 225,317 | — | — |  |
| Black | 59,758 | 0.86 | 0.83, 0.89 | <0.001 |
| Asian | 1,513 | 0.89 | 0.73, 1.09 | 0.3 |
| Other | 23,172 | 1.01 | 0.96, 1.06 | 0.7 |
| Ethnicity |  |  |  |  |
| Non-Hispanic | 293,890 | — | — |  |
| Hispanic | 15,870 | 0.83 | 0.78, 0.88 | <0.001 |
| Rurality |  |  |  |  |
| Urban | 197,548 | — | — |  |
| Rural | 112,212 | 1.05 | 1.02, 1.08 | <0.001 |
| MaritalStatus |  |  |  |  |
| Unmarried | 158,031 | — | — |  |
| Married | 151,729 | 0.92 | 0.89, 0.94 | <0.001 |
| CharlsonScore | 309,760 | 1.12 | 1.11, 1.12 | <0.001 |
| DRGWeight | 309,760 | 1.11 | 1.11, 1.12 | <0.001 |
| HospitalDays | 309,760 | 1.01 | 1.01, 1.01 | <0.001 |
| ICUDays | 309,760 | 1.00 | 1.00, 1.01 | <0.001 |
| ADINationalRank | 309,760 | 1.00 | 1.00, 1.00 | <0.001 |
| VAAdmit | 309,760 | 1.17 | 1.14, 1.20 | <0.001 |
| ^1^HR = Subdistribution Hazard Ratio, CI = Confidence Interval  Estimates are from Cox proportional hazards models with 90-day follow-up, evaluating the association between admission location (VA vs community[CCN]) and risk of mortality within 90 days of discharge. Hazard ratios (HRs) greater than 1 indicate a higher risk of death. Covariates include demographic characteristics, comorbidities, hospital and intensive care unit (ICU) length of stay, Diagnosis-Related Group (DRG) weight, Area Deprivation Index (ADI) national rank, and marital status. Reference categories are denoted by em dashes (—). | | | | |

# eTable 10. Sensitivity Analysis of Confidence Intervals for Calculated Risks Using Fine-Gray Model with M-out-of-N Nonparametric Bootstrapping

| Risk | Primary Care | Emergency Dept. | Readmission | Death |
| --- | --- | --- | --- | --- |
| **30 Day Risk** | | | | |
| Abs. Risk VA | 20.18 (19.76, 21.15) | 16.50 (16.16, 17.30) | 15.71 (15.05, 16.24) | 4.02 (3.63, 4.32) |
| Abs. Risk CCN | 15.29 (14.97, 15.95) | 21.00 (20.72, 21.87) | 14.11 (13.67, 14.66) | 3.47 (3.23, 3.74) |
| Risk Difference | 4.90 (4.24, 5.85) | -4.50 (-5.31, -3.80) | 1.60 (0.71, 2.17) | 0.55 (0.10, 0.88) |
| Risk Ratio | 1.32 (1.27, 1.39) | 0.79 (0.76, 0.82) | 1.11 (1.05, 1.16) | 1.16 (1.03, 1.26) |
| **90 Day Risk** | | | | |
| Abs. Risk VA | 28.09 (27.40, 29.12) | 25.65 (24.93, 26.55) | 27.00 (26.01, 27.73) | 8.44 (7.76, 8.93) |
| Abs. Risk CCN | 21.55 (21.01, 22.16) | 32.12 (31.57, 32.93) | 24.44 (23.86, 25.17) | 7.31 (6.95, 7.75) |
| Risk Difference | 6.54 (5.63, 7.75) | -6.47 (-7.55, -5.42) | 2.56 (1.13, 3.46) | 1.13 (0.21, 1.77) |
| Risk Ratio | 1.30 (1.26, 1.36) | 0.80 (0.77, 0.83) | 1.10 (1.05, 1.14) | 1.15 (1.03, 1.25) |
| Confidence intervals were generated using M-out-of-N nonparametric bootstrapping with 30,000 random observations for each bootstrap, and 1,000 bootstraps were performed to ensure robust estimates. The analysis was conducted for four outcomes: primary care provider (PCP) visits, emergency department (ED) visits, hospital readmissions, and deaths. For each outcome, absolute risks for Veterans Affairs (VA) and Community Care Network (CCN) discharges are presented, along with the risk difference (VA minus CCN) and risk ratio (VA/CCN). Results are provided for both 30-day and 90-day post-discharge periods. Point estimates are derived from the original fit model on the full data set. | | | | |

# eTable 11. Sensitivity of Subdistribution Hazard Ratios to Exclusion of Hospital and ICU Length of Stay Covariates

|  | Primary | Model 2 | | | Model 3 | | |
| --- | --- | --- | --- | --- | --- | --- | --- |
| **Outcome** | SHR | SHR | Diff. | % Change | SHR | Diff. | % Change |
| PCP | 1.36 | 1.34 | -0.02 | -1.49 | 1.34 | -0.02 | -1.49 |
| Emergency Dept. | 0.76 | 0.77 | 0.01 | 1.30 | 0.77 | 0.01 | 1.30 |
| Readmissions | 1.12 | 1.16 | 0.04 | 3.45 | 1.15 | 0.03 | 2.61 |
| Death | 1.17 | 1.22 | 0.05 | 4.10 | 1.21 | 0.04 | 3.31 |
| The Primary Model presents subdistribution hazard ratios (SHR) from the full Fine–Gray models specified in the manuscript, estimating the association of VA versus CCN admission location with each outcome. Model 2 replicates the primary model but excludes hospital length of stay (LOS) as a covariate. Model 3 replicates the primary model but excludes both hospital LOS and ICU LOS as covariates. For Models 2 and 3, absolute differences (Δ) and percentage changes are calculated relative to the SHR from the Primary Model. Negative values indicate a lower SHR after exclusion of LOS covariates, while positive values indicate a higher SHR. | | | | | | | |

# eTable 12. Bayesian Mediation Analysis (Absolute Risk Difference in %)

| Effect | Primary Model  (Mean, 95% CrI^1^) | Sensitivity A  (Mean, 95% CrI^1^) | Sensitivity B  (Mean, 95% CrI^1^) | Sensitivity C  (Mean, 95% CrI^1^) |
| --- | --- | --- | --- | --- |
| PNDE^2^ | 3.28 (2.58, 3.98) | 3.34 (2.65, 4.01) | 3.32 (2.63, 4.01) | 3.14 (2.06, 4.26) |
| NIE_0_ (CCN indexed)^3^ | –0.28 (–0.36, –0.22) | — | — | -0.21 (-0.31, -0.12) |
| NIE_1_ (VA indexed)^4^ | –0.20 (–0.27, –0.14) | –0.24 (–0.30, –0.19) | –0.24 (–0.29, –0.18) | -0.12 (-0.20, -0.03) |
| TE^5^ | 3.08 (2.38, 3.77) | 3.07 (2.39, 3.75) | 3.06 (2.37, 3.75) | 3.03 (1.94, 4.15) |
| ^1^CrI = credible interval. ^2^PNDE (Pure Natural Direct Effect): effect of hospital type on readmission, assuming the chance of PCP follow-up were fixed to the level seen in community hospital patients. ^3^NIE (Natural Indirect Effect): effect of differences in PCP follow-up rates on readmission. When the model includes a hospital type × PCP follow-up interaction, the NIE can differ depending on which group is used as the reference (‘CCN indexed’ vs ‘VA indexed’). In models without the interaction, both versions are identical, so only one NIE is reported. ^4^TE (Total Effect): overall effect of VA vs community admission (approximately PNDE + NIE).  Sensitivity A: model without the interaction term. Sensitivity B: excluding ICU length of stay. Sensitivity C: interaction model with wider priors. | | | | |

# eTable 13. Posterior Regression Estimates and Convergence Diagnostics for Mediator (PCP30) and Outcome (Readmit90) Models

| Parameter | Mean (95% CrI^1^) | R-hat | Bulk ESS^2^ | Tail ESS^2^ |
| --- | --- | --- | --- | --- |
| pcp30_Intercept | -1.44 (-1.52, -1.35) | 1.00 | 6527 | 5013 |
| readmit90_Intercept | -2.16 (-2.23, -2.08) | 1.00 | 6837 | 5132 |
| pcp30_VAAdmitVA | 0.45 (0.36, 0.53) | 1.01 | 450 | 1104 |
| pcp30_AdmitAge | -0.00 (-0.00, -0.00) | 1.00 | 7227 | 4898 |
| pcp30_GenderF | 0.00 (-0.06, 0.06) | 1.00 | 10840 | 4045 |
| pcp30_RaceBlack | -0.07 (-0.11, -0.04) | 1.00 | 8984 | 4177 |
| pcp30_RaceAsian | -0.11 (-0.27, 0.04) | 1.00 | 10492 | 4239 |
| pcp30_RaceOther | -0.02 (-0.07, 0.03) | 1.00 | 9680 | 3129 |
| pcp30_EthnicityHispanic | -0.01 (-0.07, 0.05) | 1.00 | 8149 | 4209 |
| pcp30_CharlsonScore | -0.00 (-0.01, 0.00) | 1.00 | 14034 | 4371 |
| pcp30_DRGWeight | -0.08 (-0.10, -0.06) | 1.00 | 9717 | 4564 |
| pcp30_rural1 | 0.03 (0.00, 0.06) | 1.00 | 8564 | 4717 |
| pcp30_MaritalStatusWidowed | 0.00 (-0.03, 0.04) | 1.00 | 10232 | 4172 |
| pcp30_MaritalStatusOther | -0.15 (-0.18, -0.12) | 1.00 | 10854 | 4725 |
| pcp30_ICUDays | -0.00 (-0.00, 0.00) | 1.00 | 8953 | 4149 |
| pcp30_ADINationalRank | -0.00 (-0.00, -0.00) | 1.00 | 6881 | 4875 |
| readmit90_VAAdmitVA | 0.16 (0.13, 0.20) | 1.00 | 1828 | 3228 |
| readmit90_pcp_30 | -0.26 (-0.30, -0.22) | 1.00 | 6255 | 4991 |
| readmit90_AdmitAge | 0.01 (0.00, 0.01) | 1.00 | 7189 | 4891 |
| readmit90_GenderF | -0.09 (-0.15, -0.04) | 1.00 | 9575 | 3275 |
| readmit90_RaceBlack | -0.07 (-0.09, -0.04) | 1.00 | 9797 | 4657 |
| readmit90_RaceAsian | -0.07 (-0.21, 0.07) | 1.00 | 10292 | 4290 |
| readmit90_RaceOther | -0.06 (-0.10, -0.02) | 1.00 | 10186 | 4110 |
| readmit90_EthnicityHispanic | -0.07 (-0.12, -0.02) | 1.00 | 8724 | 4850 |
| readmit90_CharlsonScore | 0.10 (0.09, 0.10) | 1.00 | 15332 | 4363 |
| readmit90_DRGWeight | 0.10 (0.09, 0.12) | 1.00 | 9319 | 4361 |
| readmit90_rural1 | -0.07 (-0.09, -0.05) | 1.00 | 9510 | 4299 |
| readmit90_MaritalStatusWidowed | 0.12 (0.08, 0.15) | 1.00 | 11098 | 4019 |
| readmit90_MaritalStatusOther | 0.13 (0.10, 0.16) | 1.00 | 9786 | 4500 |
| readmit90_ICUDays | 0.02 (0.02, 0.02) | 1.00 | 10239 | 5107 |
| readmit90_ADINationalRank | 0.00 (0.00, 0.00) | 1.00 | 6114 | 5119 |
| readmit90_VAAdmitVA:pcp_30 | 0.10 (0.04, 0.15) | 1.00 | 5896 | 4836 |
| ^1^CrI = credible interval; ^2^ESS = effective sample size. Coefficients are posterior means on the log-odds scale under a logit link; positive values indicate a higher probability of the event. All estimates are marginal to hospital-level random intercepts. The outcome model includes a VAAdmit × PCP30 interaction. | | | | |

# eTable 14. Distribution and Overrepresentation of DRGs and Clusters in VAMC vs. CCN Discharges

| DRG / Variant | Overall Cohort Proportion | CCN  Proportion | CCN  Global OI | CCN  within DRG OI | VAMC  Proportion | VAMC  Global OI | VAMC  within DRG OI |
| --- | --- | --- | --- | --- | --- | --- | --- |
| **AMI (All)** | 0.33 | 0.69 | 1.04 | 1.00 | 0.31 | 0.92 | 1.00 |
| Delta AMI | 0.30 | 0.63 | 0.94 | 0.91 | 0.37 | 1.11 | 1.21 |
| Omicron AMI | 0.37 | 0.63 | 0.95 | 0.91 | 0.37 | 1.11 | 1.21 |
| Gamma AMI | 0.35 | 0.62 | 0.93 | 0.89 | 0.38 | 1.13 | 1.24 |
| Epsilon AMI | 0.32 | 0.67 | 1.01 | 0.97 | 0.33 | 0.98 | 1.07 |
| Sigma AMI | 0.32 | 0.72 | 1.08 | 1.04 | 0.28 | 0.84 | 0.92 |
| **Respiratory (All)** | 0.34 | 0.61 | 0.91 | 1.00 | 0.39 | 1.18 | 1.00 |
| Delta Resp. | 0.33 | 0.49 | 0.74 | 0.82 | 0.51 | 1.51 | 1.28 |
| Omicron Resp. | 0.33 | 0.54 | 0.82 | 0.89 | 0.46 | 1.37 | 1.16 |
| Gamma Resp. | 0.32 | 0.54 | 0.81 | 0.89 | 0.46 | 1.37 | 1.17 |
| Epsilon Resp. | 0.37 | 0.55 | 0.83 | 0.91 | 0.45 | 1.35 | 1.15 |
| Sigma Resp. | 0.34 | 0.64 | 0.96 | 1.06 | 0.36 | 1.07 | 0.91 |
| **Sepsis (All)** | 0.34 | 0.70 | 1.05 | 1.00 | 0.30 | 0.90 | 1.00 |
| Delta Sepsis | 0.37 | 0.61 | 0.92 | 0.88 | 0.39 | 1.16 | 1.29 |
| Omicron Sepsis | 0.30 | 0.58 | 0.88 | 0.84 | 0.42 | 1.25 | 1.38 |
| Gamma Sepsis | 0.32 | 0.62 | 0.94 | 0.90 | 0.38 | 1.12 | 1.24 |
| Epsilon Sepsis | 0.30 | 0.66 | 0.99 | 0.94 | 0.34 | 1.02 | 1.13 |
| Sigma Sepsis | 0.34 | 0.73 | 1.10 | 1.05 | 0.27 | 0.81 | 0.89 |
| This table presents the Overrepresentation Index (OI) for various Diagnosis-Related Groups (DRGs) across different locations and clusters. The OI is a relative measure, calculated as the ratio of two proportions: the group proportion (e.g., admissions for sepsis to the VA) to the parent group proportion (e.g., total admissions to the VA). An OI value greater than 1 indicates that the condition is more prevalent than expected based on the overall distribution of patients.  The Global OI quantifies how frequently each DRG–variant combination appears at a given location relative to that location’s overall share of admissions. The denominators for the Global OI are the overall proportion of community admissions (0.66), and VA admissions (0.34). The Within DRG OI assesses the frequency of each DRG–variant combination at a location relative to its distribution among all patients with the same DRG. The denominators for the Within DRG OI are given in the diagnosis-specific "All" rows (e.g., Respiratory (All)) under CCN Proportion or VA Proportion for each diagnosis group.  DRG Definitions: Sepsis (870–872); Acute respiratory failure (003, 004, 189–195, 207, 208); Acute myocardial infarction (223–225, 280–285, 291–293) | | | | | | | |

# eFigure1. Simplified Directed Acyclic Graph Frameworks Informing Covariate Selection and Sensitivity Analyses.

(Left) *Confounding-only specification*: adjustment set includes baseline demographic, comorbidity, socioeconomic, and case mix variables (age, sex, race/ethnicity, Area Deprivation Index, year, Charlson Comorbidity Index, DRG/Case Mix Index), representing potential confounders of the relationship between hospital type (exposure) and outcomes.

(Middle) *Mediator/collider specification*: ICU and hospital length of stay are shown as potential mediators or colliders influenced by both hospital type and acute severity. Adjustment for these variables could introduce mediator or collider bias.

(Right) *Conditioned sensitivity specification*: ICU and hospital length of stay are explicitly treated as conditioned variables in sensitivity analyses. Results excluding these variables were directionally and statistically consistent, supporting the robustness of our findings.


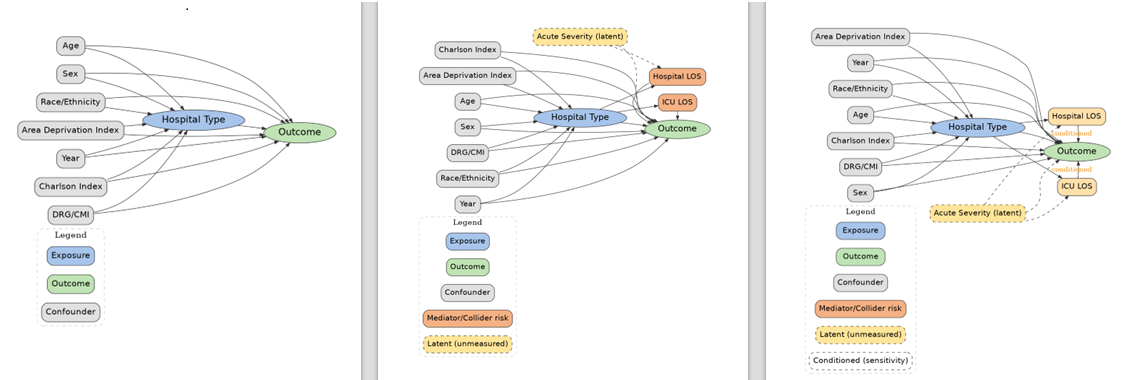


# eFigure 2. Cohort Selection Flowchart for ICU Survivors Discharged Home Between 2016 and 2023.

This figure illustrates the cohort selection process, detailing inclusion and exclusion criteria. Veterans were identified based on ICU admissions at VA and community hospitals, with exclusions for surgical admissions, non-home discharges, and missing discharge diagnosis-related group (DRG) codes. The final cohort included 311,224 veterans with post-discharge follow-up through March 2024.


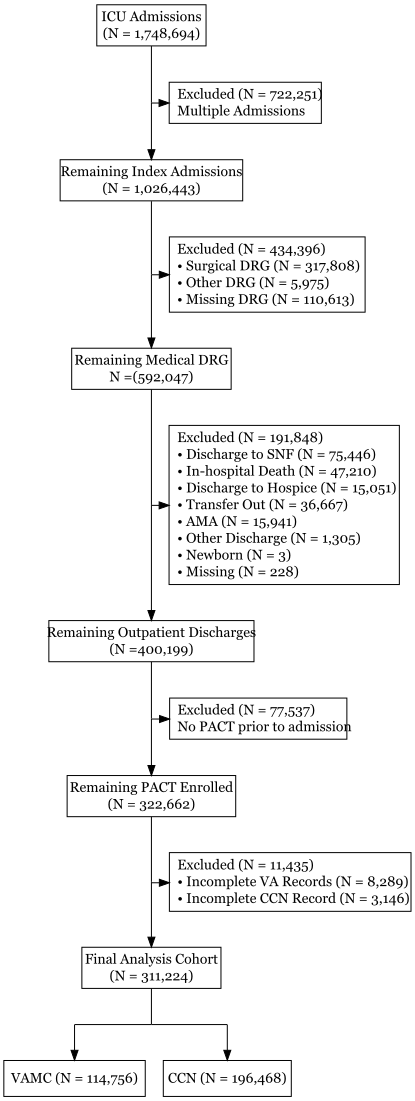


# eFigure 3. MCMC Trace Plots for Representative Mediator (PCP30) and Outcome (Readmit90) Parameters


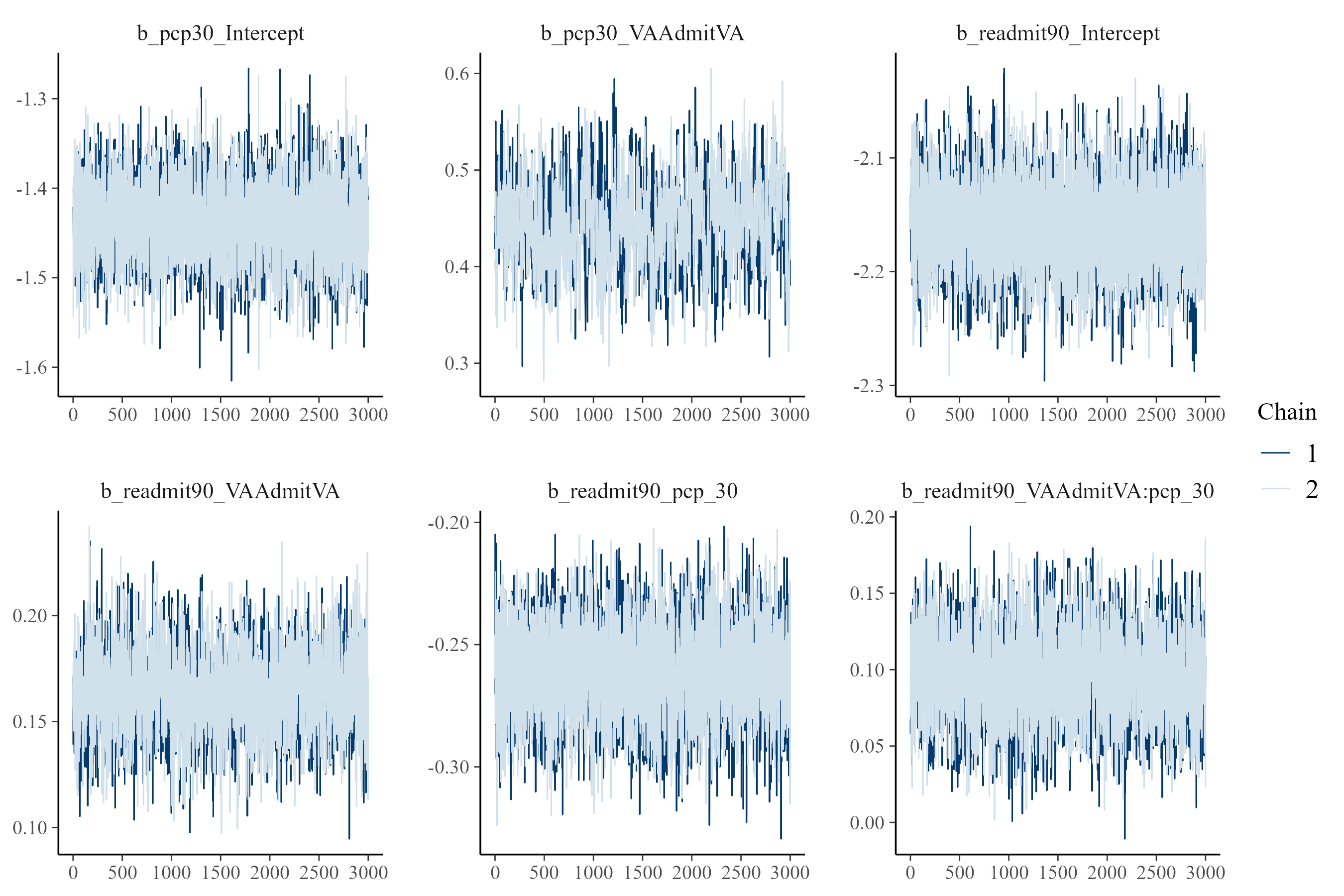


Each panel shows two independent Markov Chain Monte Carlo (MCMC) chains (dark/light) for a single parameter across post-warmup iterations (x-axis) with the sampled log-odds values on the y-axis. A dense, horizontal ‘fuzzy band’ with the chains overlapping and no sustained drift indicates good mixing and convergence to a stable posterior. The reported R-hat ≈ 1.00 and large effective sample sizes (ESS) quantify this convergence (values in eTable 9)

# eFigure 4. Decile-Binned Calibration of Predicted vs. Observed 90-day Readmission Probabilities


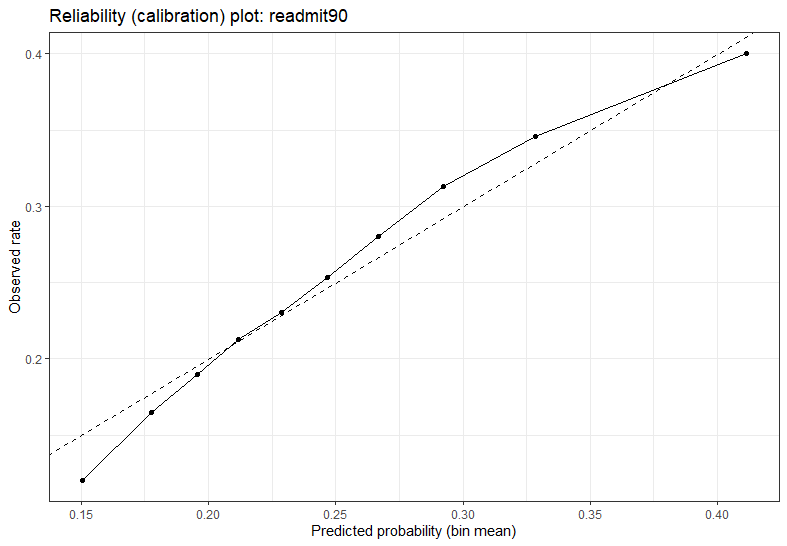


The calibration plot bins patients by predicted risk (deciles). Each point is the observed event rate vs the mean predicted probability for that bin; the dashed 45° line denotes perfect agreement. Proximity to the line indicates good calibration; systematic deviations above/below imply under/overprediction.
